# Nitrate Removal by Donnan Dialysis and Anion-Exchange Membrane Bioreactor Using Upcycled End-of-Life Reverse Osmosis Membranes

**DOI:** 10.3390/membranes12020101

**Published:** 2022-01-18

**Authors:** Amaia Lejarazu-Larrañaga, Juan M. Ortiz, Serena Molina, Sylwin Pawlowski, Claudia F. Galinha, Vanessa Otero, Eloy García-Calvo, Svetlozar Velizarov, João G. Crespo

**Affiliations:** 1 IMDEA Water Institute, Avenida Punto Com, 2, Alcalá de Henares, 28805 Madrid, Spain; juanma.ortiz@imdea.org (J.M.O.); eloy.garcia@imdea.org (E.G.-C.); 2 Chemical Engineering Department, University of Alcalá, Ctra. Madrid-Barcelona Km 33.600, Alcalá de Henares, 28871 Madrid, Spain; 3 LAQV-REQUIMTE, Department of Chemistry, NOVA School of Science and Technology, FCT NOVA, Universidade NOVA de Lisboa, 2829-516 Caparica, Portugal; s.pawlowski@fct.unl.pt (S.P.); cf.galinha@fct.unl.pt (C.F.G.); s.velizarov@fct.unl.pt (S.V.); jgc@fct.unl.pt (J.G.C.); 4 LAQV-REQUIMTE, Department of Conservation and Restoration, NOVA School of Science and Technology, FCT NOVA, Universidade NOVA de Lisboa, 2829-516 Caparica, Portugal; van_otero@campus.fct.unl.pt; 5 VICARTE, Department of Conservation and Restoration, NOVA School of Science and Technology, FCT NOVA, Universidade NOVA de Lisboa, 2829-516 Caparica, Portugal

**Keywords:** membrane recycling, nitrate removal, Donnan Dialysis, Ion-Exchange Membrane Bioreactor, circular economy

## Abstract

This work explores the application of Reverse Osmosis (RO) upcycled membranes, as Anion Exchange Membranes (AEMs) in Donnan Dialysis (DD) and related processes, such as the Ion Exchange Membrane Bioreactor (IEMB), for the removal of nitrate from contaminated water, to meet drinking water standards. Such upcycled membranes might be manufactured at a lower price than commercial AEMs, while their utilization reinforces the commitment to a circular economy transition. In an effort to gain a better understanding of such AEMs, confocal µ-Raman spectroscopy was employed, to assess the distribution of the ion-exchange sites through the thickness of the prepared membranes, and 2D fluorescence spectroscopy, to evaluate alterations in the membranes caused by fouling and chemical cleaning The best performing membrane reached a 56% average nitrate removal within 24 h in the DD and IEMB systems, with the latter furthermore allowing for simultaneous elimination of the pollutant by biological denitrification, thus avoiding its discharge into the environment. Overall, this work validates the technical feasibility of using RO upcycled AEMs in DD and IEMB processes for nitrate removal. This membrane recycling concept might also find applications for the removal and/or recovery of other target negatively charged species.

## 1. Introduction

Reverse Osmosis (RO) desalination market is in constant growth [1]. However, due to RO performance deterioration, an annual membrane replacement rate ranging between 5% in brackish water RO desalination to 20% in sea water RO desalination is estimated [2]. To date, end-of-life RO membranes are commonly wasted in landfills, generating an increasing environmental concern [3]. Membrane recycling is a more sustainable alternative, and hence, it is gaining an increasing attention by researchers and industry stakeholders. Recently, the upcycling of end-of-life RO membranes for the preparation of Anion-Exchange Membranes (AEMs) has been proposed [4,5,6]. This alternative entails the deconstruction of the RO module, allowing for the individual management of flat sheet membranes and plastic components. The resulting membranes have a good permselectivity but a relatively high electrical resistance, which increases the energy consumption associated with their use in electrodialysis (ED) [4]. In addition, ED necessarily removes both cations and anions (i.e., leading to demineralization), and it might be less appropriate in certain applications, such as drinking water or beverages production, in which the ionic composition should be preserved as much as possible. In that respect, an electrochemical potential gradient driven process, such as Donnan Dialysis (DD) and its integration with biotransformation, referred to as Ion-Exchange Membrane Bioreactor (IEMB), could entail a more suitable application for such RO upcycled AEMs. Since DD, utilizes counter-diffusion of two or more ions through an ion-exchange membrane to achieve their separation, its energy demand is much lower in comparison to that of ED [7]. Indeed, owing to its simplicity of installation and low operating costs (under batch operation conditions DD can be operated even without pumping), DD has been presented as an appropriated technology for household water treatment in remote rural areas of developing countries (not connected to centralized water supply facilities) [8,9]. Furthermore, the IEMB has been reported as an efficient technology for removal and degradation of target ionic pollutants, such as nitrate, from drinking water supplies [10].

Nitrate is toxic for human health. The abusive use of nitrogen-rich fertilizers, uncontrolled sludge and slurry dumping, and a poor wastewater treatment (i.e., an uncompleted biological denitrification process), have risen its concentration in natural waters (specially in groundwaters) to critical levels worldwide [11,12]. The World Health Organization (WHO) has limited its concentration to a maximum of 50 mg L^−1^ in drinking water, along with the recommendation to keep it below 25 mg L^−1^ [13]. However, in drinking water production, nitrate is not eliminated by regularly used technologies (i.e., coagulation-flocculation, decantation, filtration, and disinfection), and frequently other methods, such as RO [14], ED [15], ion-exchange resins [16], and biological denitrification [17], have been employed for this purpose. These processes have several disadvantages such as: high economic and energy costs, the extensive use of chemicals, or the inception of secondary pollution problems in the treated water (biological contamination) [18].

For the removal of nitrate by DD, an anion-exchange membrane (AEM) is used to separate the feed solution (i.e., water polluted with nitrate), from the receiver solution containing a higher concentration of a “driving” nontoxic counter-ion (chloride, in this work). In such system, chloride is transported through the AEM to the diluted nitrate-containing solution by diffusion, due to its concentration gradient. As the AEM rejects the co-ions, an electrochemical potential gradient is established across the membrane, causing the coupled back-transport of the target counter ion (nitrate, in this work) to the receiver solution [7]. The continuous diffusive transport of the driving counter ion to the diluted compartment eventually leads to the transport of nitrate against its concentration gradient (uphill transport), thus allowing an almost complete removal of the target ionic pollutant from water and its consequent concentration in the receiver compartment. Hence, a post treatment is needed for the elimination of the nitrate accumulated in the receiver compartment. To overcome this issue, the IEMB combines the DD principle with a biological conversion process in the receiver compartment, providing the simultaneous transport and elimination of nitrate by conversion to molecular nitrogen, in a single device and in a single step [10]. In the bioreactor, a microbial culture of denitrifying bacteria oxidizes a carbon source (the electron donor) added to the receiver. In the absence of oxygen (anoxic/anaerobic environment), the nitrate acts as the electron acceptor, and it is sequentially reduced to NO_2_, NO, N_2_O, and finally N_2_ [19]. Furthermore, the integration of a membrane in the bioreactor allows the physical separation of the microbial culture and other pollutants from the treated water (e.g., residual carbon source and by-products from microbial metabolism), thus preventing the major problems associated to the application of biological treatments to drinking water production [20]. In previous studies, IEMB process achieved a high efficiency in drinking water denitrification when using Neosepta ACS membrane [21]. However, the high cost of such membrane could compromise the practical implementation of the IEMB concept, and thus, the use of more affordable membranes is highly desirable [22]. In this context, the AEMs prepared from upcycled RO membranes could entail a more economic approach.

In the present work, the use of AEMs upcycled from end-of-life RO membranes in DD and IEMB processes for the removal of nitrate from a synthetic polluted water was studied and compared with the performance of a Ralex ^®^ AMH-PES (a commercial AEM manufactured by MemBrain ^®^ s.r.o., Stráž pod Ralskem, Czech Republic) selected due to its high durability, good mechanical and chemical stability and a relatively low commercial cost. Further, the membrane structure was analysed by confocal µ-Raman spectroscopy, and alterations in the membranes due to fouling and the chemical cleaning were evaluated by 2D fluorescence spectroscopy. Finally, a preliminary estimation of the material cost to manufacture the upcycled membranes was performed.

## 2. Materials and Methods

### 2.1. Chemical Reagents

Sodium hypochlorite (NaClO, 14%), tetrahydrofuran (THF), sodium sulphate (Na_2_SO_4_), and ammonium chloride (NH_4_Cl) were purchased from Scharlab, S.L., Barcelona, Spain. Sodium chloride (NaCl), sodium nitrate (NaNO_3_), di-potassium hydrogen phosphate anhydrous (K_2_HPO_4_), humic acids (HA), Amberlite^®^ IRA-402, and Lewatit ^®^ Sybron Ionac ^®^ SR-7 anion-exchange resins were supplied by Merck KGaA, Darmstadt, Germany. Puroliteº^®^ A600/9413 anion-exchange resin was supplied by MemBrain ^®^ s.r.o., Stráž pod Ralskem, Czech Republic. Potassium dihydrogen phosphate (KH_2_PO_4_) was purchased from Chem-lab NV, Zedelgem, Belgium; sodium hydrogen phosphate (NaH_2_PO_4_) from Panreac Química S.L.U., Barcelona, Spain; magnesium sulphate heptahydrate (MgSO_4_·7H_2_O) from LabChem Inc., Zelienople, PA, USA; sodium hydroxide (NaOH) from Fisher Scientific Co. LLC., Pittsburgh, PA, USA, and citric acid from VWR chemicals, USA. Polyvinylchloride (PVC, Mw = 112,000 g mol^−1^) was supplied by ATOCHEM, Madrid, Spain. MilliQ water was used throughout the experiments.

### 2.2. Membranes

**Figure 1:**
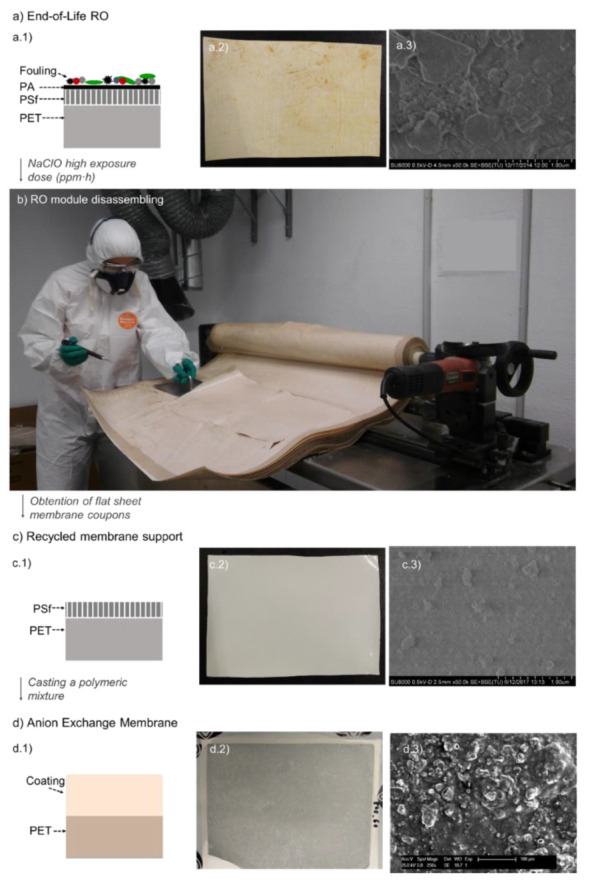
Steps for the preparation of RO upcycled Anion Exchange Membranes: (**a**) End-of-life RO membrane, (**a1**) schematic representation, (**a2**), surface image, (**a3**) surface SEM image. (**b**) Disassembled end-of-life RO module and separation of flat sheet membrane coupons; (**c**) recycled membrane support, (**c1**) schematic representation, (**c2**) surface image, (**c3**) surface SEM image. (**d**) Prepared AEM (**d1**) schematic representation, (**d2**), surface image, (**d3**) surface SEM image. PA, polyamide; PSf, Polysulfone, PET, polyester.

Figure 1. illustrates the different steps carried out for the preparation of the RO upcycled AEMs. To complement this representation, Scanning Electron Microscopy (SEM) images of the membranes are included in Figure 1(a3,c3,d3). Due to the morphological differences between the surfaces of the membranes, different microscope magnifications were employed. The methodology for conducting the SEM analyses was described elsewhere [4,23]. An end-of-life thin film composite polyamide RO membrane (TM 720-400, Toray Industries, Inc., Osaka, Japan), discarded by a desalination plant after more than three years of exploitation, was upcycled as mechanical support to prepare AEMs (Figure 1a) [4]. First of all, the end-of-life RO module was treated by passive immersion in 9000 mg L^−1^ NaClO solution for 90 h, as described in [24,25]. Then, the spiral wound configuration of the module was opened (module autopsy), using a radial arm saw, and the membrane sheets were unrolled (Figure 1b). Membrane coupons of approximately 315 cm^2^ were then taken out to be used as mechanical support in the preparation of AEMs. The high exposure dose of NaClO employed in the previous step (810,000 ppm·h) ensured the complete elimination of the fouling and of the polyamide (PA) layer, and leaded to a considerably homogeneous and microporous polysulfone (PSf) surface (Figure 1c), as it was demonstrated in previous studies carried out by our research group [26,27,28]. Thus, the membranes achieved ultrafiltration (UF)-like properties in terms of salt rejection and hydraulic permeability [26]. Afterwards, the AEMs were prepared by casting a polymeric mixture on the polysulfone (PSf) surface of the recycled membrane (Figure 1d). The polymeric mixture contained PVC dissolved into THF and a finely grounded anion-exchange resin (sieved down to less than 50 µm), in a blend ratio of 0.5 g:0.5 g:10 mL, respectively, ion-exchange resin (g) to PVC (g), to THF (mL). The solvent was evaporated at room temperature for 1 h, and then, the membrane was immersed in a water bath. Three different types of anion-exchange resins were tested: Purolite^®^ A600/9149, Amberlite^®^ IRA-402 and Lewatit^®^ Sybron Ionac^®^ SR-7 (see Table 1). The resulting membranes were named as Pur-RE, Amb-RE, and Lew-RE, henceforth. The methodology for the preparation of the membranes is described in more detail in [4]. The microstructure of such membranes was deeply characterized before [4,6] (see Figure 1(d3), and it is further analysed by confocal µ-Raman spectroscopy in the present work (Section 3.1). The prepared membranes show a considerably uniform distribution of the ion-exchange resin at the membrane surface, having a heterogeneous microstructure due to the presence of conductive (ion-exchange resin particles) and non-conductive regions (PVC, PET, and PSf). Therefore, a commercial membrane with a heterogeneous structure was used as reference membrane, in this case, Ralex ^®^ AMH-PES from Mega a.s., which was selected due to its durability, high mechanical stability and relatively low commercial cost.

### 2.3. Confocal Micro-Raman Spectroscopy

In this work, confocal µ-Raman spectroscopy was used to analyse the distribution of the polymeric composition and the ion-exchange sites, along the thickness of the Pur-RE membrane. Since the same membrane preparation methodology was employed to prepare all the membranes (Pure-RE, Amb-RE and Lew-RE) and only the type of the ion exchange resin was different, analogous structures are expected in all the prepared membranes. To assess that structure, Pur-RE membrane was selected for confocal micro-Raman spectroscopy characterisation since that membrane showed the best performance under DD and IEMB operating conditions (results shown in Section 3.2). A Labram 300 Jobin Yvon spectrometer from Horiba Ltd., Japan, was used, equipped with a solid-state laser operating at 532 nm. The laser beam was focused with a 50 × Olympus objective lens. The analysis was performed with a neutral density filter of 0.6 optical density, with 15-s exposure time and 20 scans. Spectra were recorded as an extended scan and presented with a break between 1025 and 1085 cm^−1^ to remove the contribution of bands attributed to nitrate compounds. For obtaining the cross-section, the membrane was frozen into liquid nitrogen and broken properly. The analyses were performed in 10 consecutive points in a row across the membrane section, from the coated side to the PET side of the membrane. The distance between the analysed points was about 20 µm.

### 2.4. Donnan Dialysis (DD) and Ion-Exchange Membrane Bioreactor (IEMB) Experiments

#### 2.4.1. Model Solutions, Test Cell, and Analytical Methods

The model polluted water, referred as feed henceforth, consisted of deionized water supplemented with 80 mg L^−1^ of nitrate (added as NaNO_3_). This concentration was selected as a model high nitrate concentration (>50 mg L^−1^), as it was used before in [30]. The receiver aqueous solution contained chloride as a major counter-ion (3545 mg L^−1^ of Cl^−^, added as NaCl) and a mixture of inorganic nutrients (1000 mg L^−1^ K_2_HPO_4_, 592 mg L^−1^ KH_2_PO_4_, 500 mg L^−1^ NaH_2_PO_4_, 233 mg L^−1^ NH_4_Cl, and 1000 mg L^−1^ MgSO_4_·7H_2_O [31]) to support the microbial growth in the bioreactor (when operating as IEMB). In order to maintain the same ionic strength in DD and IEMB operation conditions, the nutrient enriched water media was used as a receiver solution also for DD experiments. The receiver compartment in the IEMB (referred to as biocompartment, hereafter) was additionally supplemented with microbial culture (as described in Section 2.4.3) and ethanol (at a rate of 400 mg L^−1^ day^−1^ EtOH) to ensure the presence of the carbon source in the IEMB experiments.

**Figure 2:**
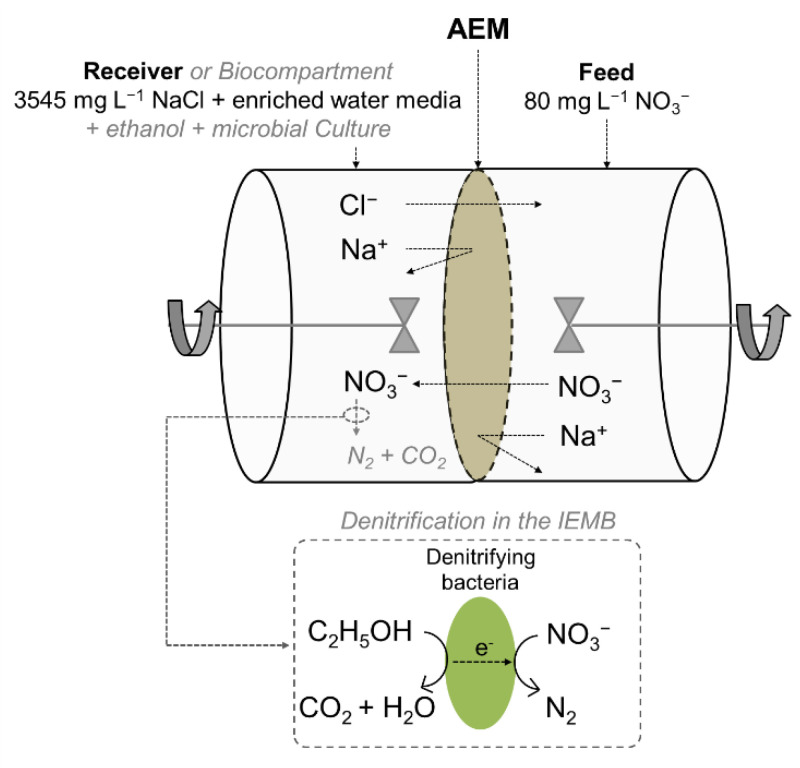
Schematic diagram of the test cell and representation of the main ionic transport in DD and IEMB experiments. Effective membrane area, 11.3 cm^2^. Volume of each compartment, 175 mL. The modifications to operate as an IEMB are represented in grey colour and italic lettering. The receiver compartment under IEMB operation was referred to as biocompartment.

The same test cell (Figure 2) was used in DD and IEMB experiments. The membrane effective area was 11.3 cm^2^ and the volume of each of the two compartments was 175 mL. The coated side of the membrane was faced to the feed compartment (polluted water). During the experiments, the solutions were continuously stirred to minimize possible concentration polarization effects. Before the experiments, all tested membranes (listed in Table 1) were equilibrated by immersion in the receiver solution (i.e., the salt mixture, without the microbial culture and ethanol) for 2 days.

The experimental time was 96 h in the case of Pur-RE, Amb-RE, and Lew-RE membranes and 72 h in the case of AMH-PES (as nitrate was already completely removed from the feed after that period). The membranes were thoroughly rinsed with water before the chemical cleaning and after each step [32].

#### 2.4.2. Operation as a Donnan Dialyzer

The time course concentration of nitrate was measured in both compartments during the experiments as described in Section 2.4.4. The membrane performance in DD operation was evaluated in terms of nitrate removal yield from the feed (%), and nitrate recovery in the receiver solution (%), as detailed in Equations (1) and (2), applicable to DD systems [33].

(1) $${\mathrm{Removal}}_{NO_{3}^{-}\left(t\right)}=\frac{C_{{\mathrm{NO}}_{3}^{-},\;\mathrm{feed}\;\left(0\right)}-{\;C}_{{\mathrm{NO}}_{3}^{-},\;\mathrm{feed}\;\left(t\right)}}{C_{{\mathrm{NO}}_{3}^{-},\;\mathrm{feed}\;\left(0\right)}}\cdot 100\%,\;$$

(2) $${\mathrm{Recovery}}_{NO_{3}^{-}\left(t\right)}=\frac{C_{{\mathrm{NO}}_{3}^{-},\;\mathrm{receiv}.\;\left(t\right)}}{C_{{\mathrm{NO}}_{3}^{-},\;\mathrm{feed}\;\left(0\right)}}\cdot 100\%$$

where $C_{NO_{3}^{-}}$ is the concentration of nitrate.

The conductivity was checked in both compartments during the experiments.

#### 2.4.3. Operation as an Ion-Exchange Membrane Bioreactor

##### Microbial Culture

The activated sludge obtained from a laboratory bioreactor fed with real wastewater was used as inoculum. The inoculum was centrifuged for 30 min at 4000 rpm, under a temperature of 4 ºC, for obtaining a pellet enriched in microorganisms and biomass. The pellet was resuspended in fresh nutrient enriched water media (composition detailed in Section 2.4.1), 800 mg L^−1^ of ethanol (i.e., the carbon source in the IEMB) and 80 mg L^−1^ of nitrate (i.e., the electron acceptor under anoxic conditions, added as NaNO_3_) were supplied to support the microbial activity and to ensure the acclimation of the bacteria to the experimental conditions. The culture was degasified with N_2_ and CO_2_ in order to preserve anaerobic conditions. Then, it was incubated at 37 ºC for 5 days. The same procedure was repeated 3 times (centrifugation, resuspension in fresh enriched water media and addition of ethanol and nitrate). After that, the enriched microbial culture was placed in fresh enriched water media and stored at 4 ºC, being ready to be used as inoculum in the IEMB.

##### Ethanol Permeation Studies

For the operation as an IEMB, it is of primary importance to avoid the permeation of the carbon source (ethanol, in this case), from the receiver solution to the feed, in order to prevent cross contamination issues (i.e., secondary pollution) in the treated water. The diffusion coefficients of ethanol across the analysed membranes were measured in a two compartments cell. (i.e., two glass bottles of 250 mL each, sealed with a lid, under stirring at 400 rpm). Each membrane under study, with an effective area of 4.52 cm^2^, was placed between the compartments. One of the compartments was filled with an aqueous ethanol solution (1000 mg L^−1^ ethanol), and the other one was filled with MilliQ water. As long as ethanol was the only organic compound in the solutions, the Total Organic Carbon (TOC) concentration was analysed in both compartments during the experimental time course (15 days) and the concentration of ethanol was calculated. Then, the diffusion coefficients of ethanol through the membranes were calculated by the graphical representation of Fick’s law Equation (3) [4].

(3) $$J_{D}=-D\cdot \frac{dC}{dl}$$

where *J_D_* (mol cm^−2^ s^−1^) is the flux of ethanol through the membrane, *D* (cm^2^ s^−1^) is the diffusion coefficient, *dC* (mol cm^−3^) is the concentration gradient (the driving force), and *dl* (cm) is the membrane thickness. In order to estimate the diffusion coefficient *D*, the linear region of the experiments was considered, where the slope of the curve C vs time has constant value, and assuming *dC* ≈ Δ*C* and *dl* ≈ *l*.

##### Operation as a Bioreactor

In the case of IEMB experiments, the receiver compartment (Figure 2) was filled with fresh enriched water media and inoculated with 15 mL of enriched microbial culture (the experimental procedure for obtaining the microbial culture has been described in this section in “*Microbial culture*” section). Before inoculation, the microbial culture was stored at room temperature for more than 48 h, for activation of the microbial metabolism. The anaerobic conditions in the receiver compartment were maintained during the experiment by continuously sparging nitrogen gas through the compartment headspace. The concentration of nitrate was measured in both compartments during the experimental time and nitrate removal yield was calculated using Equation (1). In addition, the concentration of nitrite was measured in both compartments, to verify that nitrite traces were not accumulated, ensuring that the complete bio-reduction of nitrate to nitrogen was attained. Nitrite is more toxic than nitrate, and its maximum allowed concentration is limited to 3 mg L^−1^ in drinking water [13]. In addition, the TOC in the feed (water) compartment was analysed to prove that the diffusion of ethanol from the biocompartment to the feed was avoided. The conductivity was checked in both compartments during the experiments.

#### 2.4.4. Analytical Methods

Nitrate and nitrite were analysed based on the colorimetric cadmium reduction method, according with the standard methods [34], using a Skalar SAN++ CFA analyser (Skalar Analytical B.V., Breda, The Netherlands). The TOC was measured using a TOC-V CSH total organic carbon analyser (Shimadzu Corp., Kyoto, Japan), then the corresponding concentration of ethanol was calculated by a conversion factor. The conductivity of the solutions was measured using a Sension +EC7 instruments (from Hach Company, Loveland, Colorado, United States).

### 2.5. 2D Fluorescence Spectroscopy

2D fluorescence spectroscopy has been recently proposed for in situ monitoring bioreactors and ion-exchange membrane processes [35]. In this work, 2D fluorescence spectroscopy was used to analyse alterations in the membrane surface caused by (i) their use in the bioreactor experiments (as it is described in Section 2.4.3), (ii) an accelerated organic fouling test (on pristine membranes), and (iii) chemical cleaning (of pristine membranes and those used in IEMB).

The accelerated fouling test consisted of the passive immersion of the membranes in a 100 mg L^−1^ aqueous Humic Acid (HA) solution for 1 h, at room temperature [36].

The cleaning treatment involved the passive immersion of the membranes in alkali and acid solutions, and consisted of the following steps, according to [32]:

1. Rinse with DI water
2. Passive immersion in 5000 mg L^−1^ NaOH aqueous solution for 10 min, at room temperature.
3. Rinse with DI water
4. Passive immersion in a 20,000 mg L^−1^ citric acid aqueous solution for 30 min, at room temperature.
5. Rinse with DI water

A Varian Cary Eclipse fluorescence spectrophotometer, equipped with excitation and emission monochromators, and coupled to an optical fibre bundle probe was used. The optical fibre bundle is constituted by 294 optical fibres, each with a diameter of 200 µm and a length of 2 m. The Excitation–Emission Matrices (EEMs) of the membrane surface samples were obtained between 250 and 690 nm excitation and from 260 to 700 nm emission with 5 nm step and slits of 10 and 5 nm, respectively. EEMs of both coated (top) and PET (bottom) surfaces of all membranes were acquired when pristine, after used in IEMB, after accelerated fouling test and after chemical cleaning of pristine and used in IEMB. All measurements were performed in triplicate.

Before performing a principal component analysis (PCA) of obtained EEMs, spectral data were standardized (by subtracting averages and dividing by standard deviations) for each pair of excitation emission intensity. OCTAVE GNU software was used to implement the computational routines in PCA (through PARAFAC algorithm [37]). PCA was performed by using the following two different data combinations: (1) only EEMs of the prepared membranes (Pur-RE, Amb-RE, and Lew-RE, in a total of 91 spectra); (2) EEMs of the prepared membranes and commercial membrane together (Pur-RE, Amb-RE, Lew-RE, and AMH-PES in a total of 120 spectra). PARAFAC analysis of only modified membranes captured 81.76% of the variance by the first two principal components (PCs), while PARAFAC analysis of all membranes (including commercial) captured 83.84% variance by the first two PCs.

## 3. Results

### 3.1. Confocal Micro-Raman Spectroscopy

**Figure 3:**
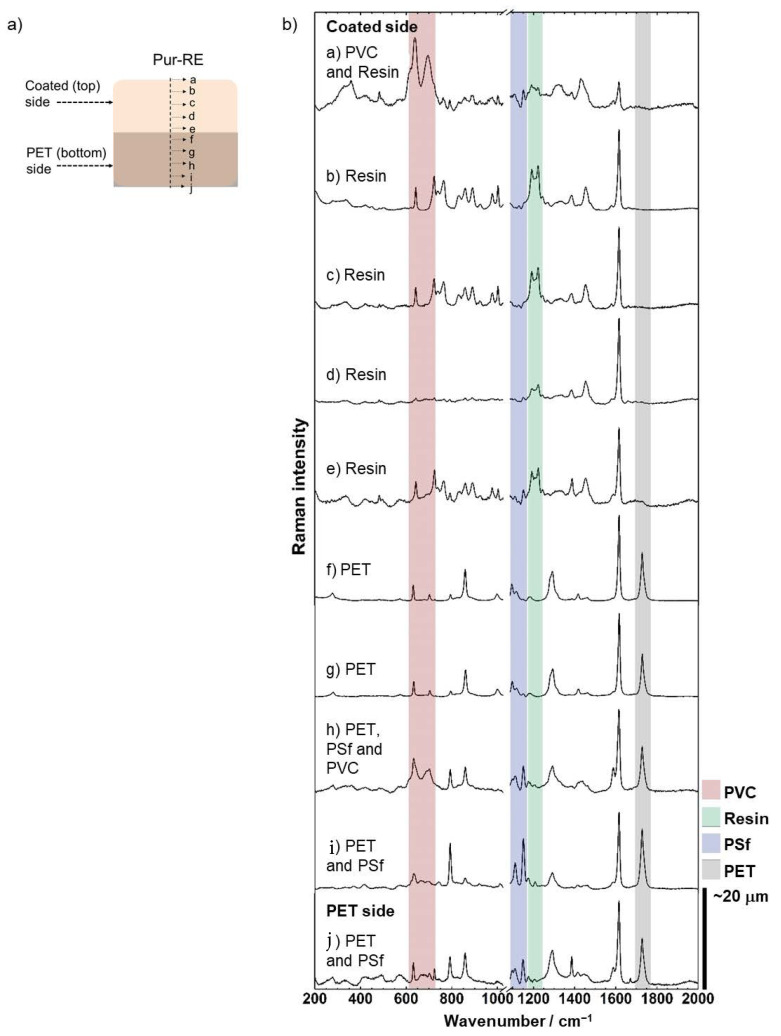
(**a**) Schematic representation of the analysed points in the membrane section. (**b**) µ-Raman spectra of the polymers in the membrane section, at the analysed points a to j. The main bands that allow for a clear identification of each polymer are highlighted.

Confocal µ-Raman spectroscopy was used to understand better the polymeric composition/structure of the cross-section membrane Pur-RE (the best performing membrane under DD and IEMB operating conditions, as discussed below). Figure 3a represents the examined locations (from a to j) along the membrane cross-section, and Figure 3b shows the Raman spectra corresponding to these locations. The main characteristic Raman bands identifying the polymers in the membrane, as well as the Raman spectra of those polymers, were characterised according to the literature [38] and are presented as Supplementary material in Table S1 and Figure S1, respectively.

At the outermost coated surface (top) of the membrane (point a), PVC is identified by its characteristic Raman bands at 638 and 695 cm^−1^ due to C-Cl stretching vibrations (highlighted with red in Figure 3b). The PVC was used as polymer binder in the coating solution, and thus, its presence in point a demonstrated an effective formation of a film on the coated membrane surface. At points a to e, the spectrum of the ion-exchange resin was found, particularly due to its characteristic Raman bands at 1190 and 1221 cm^−1^ attributed to ring C-H vibration modes (highlighted with green in Figure 3b), which verifies the successful deposition of the ion-exchange resin in the coated (top) surface of the membrane and, therefore, the presence of fixed charged functional groups. The lower intensity of the resin signal at the point a corresponds to an overshadowed effect by the PVC signal, whereas at points f to j (i.e., the PET bottom part of the membrane), the ion-exchange resin spectrum is no longer present. At these points (f to j), the spectra corresponding to PET and PSf, in different combinations, can be distinguished by their characteristic Raman bands at 1727 cm^−1^ for PET C =O stretching vibration (highlighted with grey in Figure 3b), and at 1150 and 1109 cm^−1^ for PSf C-O-C and SO_2_ stretching vibrations, respectively (highlighted with blue in Figure 3b). In the recycled membrane support, the PSf layer conforms the outermost top surface of the membrane covering the PET layer (see Figure 1b), whereas in Pur-RE, the signal corresponding to PSf was detected deeply inside the PET layer (points h to j). These results indicate that the employed organic solvent in the coating mixture (i.e., THF) has dissolved the PSf layer of the recycled membrane support, causing the percolation of dissolved PSf inside the PET layer, while the solvation of the PSf layer promotes the penetration of the polymer binder (i.e., PVC) into the recycled membrane support, as it is demonstrated by the presence of the PVC signal at point h. As a result, the adhesion of the coated layer to the recycled membrane support is strengthened, providing a high mechanical stability to the membrane [4]. In contrast, the penetration of the ion-exchange resin in the membrane support is probably limited by the size of the ion-exchange resin particles. Overall, the absence of charged functional groups in combination with the presence of non-conductive polymers in this region of the membrane (points h to j) contributes to the high electrical resistance, low IEC, and low WC of the membrane (see Table 1) [6]. In conclusion, these results confirm successful upcycling of RO membranes into AEMs and the asymmetric distribution of the fixed charged functional groups along the thickness of the PU-RE membrane. In addition, since the only difference between the prepared membranes was the type of ion-exchange resin used during the preparation, analogous results are expected for Amb-RE and Lew-RE.

### 3.2. Donnan Dialysis (DD) and Ion-Exchange Membrane Bioreactor (IEMB) Experiments

#### 3.2.1. Time Course Concentration of Nitrate in the Feed and in the Receiver Compartments

**Figure 4:**
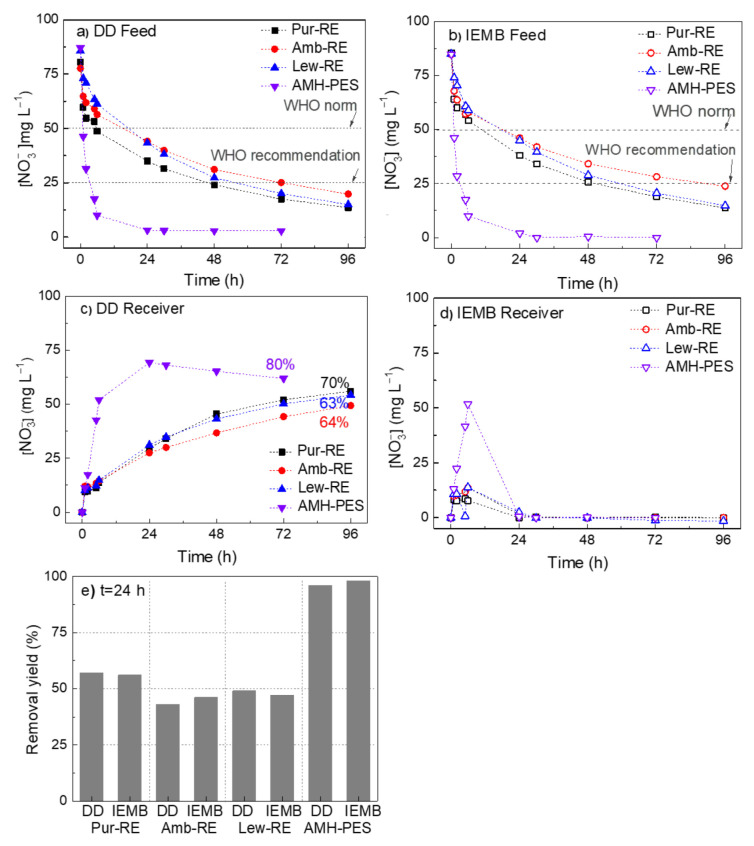
Time course concentration of nitrate ([NO_3_^−^]) in the feed compartment in (**a**) DD experiments and (**b**) IEMB experiments. Time course concentration of nitrate in the receiver compartment in (**c**) DD experiments (the numbers in % indicate the recovery yield of nitrate at the end of the experiment) and (**d**) IEMB experiments (where nitrate conversion to N_2_ occurs). (**e**) Nitrate removal yield (%) from the feed within 24 h operation as DD and IEMB. Membranes: Pur-RE, Amb-RE, Lew-RE, AMH-PES.

Figure 4 shows the time course concentration of nitrate in the feed and the receiver compartments and nitrate removal rates from the feed compartment during DD and IEMB experiments.

Figure 4a,b shows considerable differences in the performance of the membranes for reducing the concentration of the target ion to the maximum and the recommended levels (50 and 25 mg L^−1^, respectively). Such differences are correlated to the membrane properties (reported in Table 1). Accordingly, the AMH-PES membrane clearly shows the best performance, achieving 96% and 98% removal within 24 h, under DD and IEMB operation conditions, respectively (Figure 4e). This membrane has a relatively uniform distribution of the ion-exchange resin particles across the membrane section (reported before, in [4]), together with the highest IEC and WC (2.19 ± 0.09 mmol g^−1^ and 50 ± 0%, respectively), a high permselectivity (81 ± 1%) and a relatively low electrical resistance (19 ± 3 Ω cm^2^), in comparison with the prepared membranes.

In contrast, the asymmetric distribution of the ion-exchange functional groups in the prepared membranes (confirmed by µ-Raman spectroscopy, Section 3.1.) might reduce the counter-ion transport rate. Further, the relatively low ion-exchange capacity (IEC) of such membranes results in a lower number of fixed charged functional groups, meaning that a lower number of ion transport sites are available [39,40]. In addition, the low water content of the prepared membranes leads to a lower electrical conductivity [41]. As a result of the combination of the aforementioned properties, the prepared membranes have a high electrical resistance (see Table 1), as explained in detail in our previous works [4,6]. A high electrical resistance leads, under the same experimental conditions, to a slower effective transport of the ions (i.e., migration). Although, among the prepared membranes, Pur-RE shows the best performance, reaching a 57% and 56% removal within 24 h, under DD and IEMB operation conditions, respectively (Figure 4e). These results might be related to the higher IEC and the lower electrical resistance of the Pur-RE membrane (0.75 mmol g^−1^ and 56 Ω cm^2^, respectively), in comparison with the IEC and the electrical resistance of Amb-RE (0.62 mmol g^−1^ and 129 Ω cm^2^, respectively) and Lew-RE membranes (0.39 mmol g^−1^ and 120 Ω cm^2^, respectively) [20].

Figure 4c shows the accumulation of nitrate in the receiver compartment during DD experiments. Among the prepared membranes, Pur-RE membrane shows again the best performance for the recovery of nitrate under DD operation (recovery of 70%), although the performance of AMH-PES is larger (recovery of 80%). Differently, in Figure 4d, corresponding to the IEMB operation, it can be observed that the integration of a bioreactor results in the successful elimination of nitrate from the receiver compartment by denitrifying bacteria in a single device and a single step. Moreover, the time course concentration analyses of nitrite (NO_2_^−^) showed that its accumulation was avoided in both compartments (results reported as Supplementary Material in Figure S2). The latter entails several advantages such as avoiding the discharge of nitrite into the environment, while enabling a prolonged use of the receiver solution. In addition, the concentration gradient of nitrate is effectively maintained during the process, which under a continuous operation would result in favoured nitrate transport in respect to that of other accompanying anions which are not bioreduced (not evident in the present case due to batch IEMB operating conditions and the use of a single anion (NO_3_^−^) in the feed) [42].

#### 3.2.2. Co-Ion Leakage to the Feed Compartment

**Figure 5:**
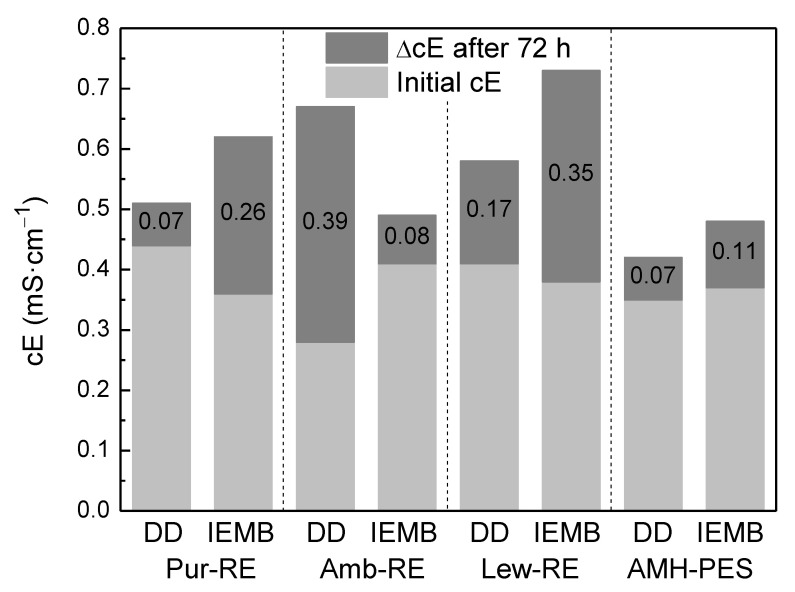
Increase of feed solution conductivity after 72 h of operation as a Donnan Dialyzer and as an IEMB.

As membranes are not 100% permselective, co-ion leakage from the receiver to the feed compartment is expected. The co-ion leakage reduces the electrochemical potential difference established across the membrane, decreasing the back-transport of the target counter-ion to the receiver compartment. Moreover, it increases the conductivity in the feed compartment. In this respect, the WHO establishes a maximum conductivity of 1 mS cm^−1^ for water intended for drinking purposes [13]. Figure 5 shows the conductivity increase in the feed compartment after 72 h of experiment.

As it can be observed in Figure 5, some co-ion leakage occurred in all experiments, resulting in a slight conductivity increase of the feed solution (e.g., a 0.07 and a 0.26 mS cm^−1^ increase using Pur-RE under DD and IEMB operating conditions, and a 0.07 and a 0.11 mS cm^−1^ increase using AMH-PES under the same conditions). However, the final conductivity in the feed compartment was maintained in all the cases below 1 mS cm^−1^. Even if the permselectivity of Pur-RE (65%) is not as high as that of AMH-PES (81%), these small increases in conductivity demonstrated that co-ion leakage is not a problem in this process [20]. Indeed, the Pur-RE membrane achieved a better performance than the Amb-RE membrane (with a permselectivity of 83%), indicating that, in this case, other membrane properties may have a greater influence in the process performance, as it was discussed in the previous subsection.

#### 3.2.3. Diffusion Permeability of the Carbon Source

**Figure 6:**
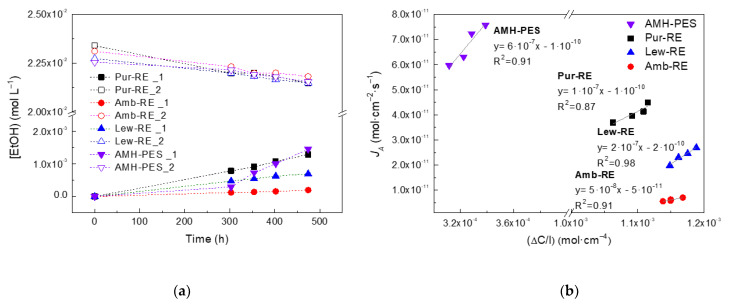
Studies on ethanol permeation across the membranes. (**a**) Time course concentration change in the water compartment (1) and the ethanol compartment (2), (**b**) representation used for calculation of ethanol diffusion coefficients across the membranes and linear equations for each membrane.

The migration of the carbon source from the receiver to the feed compartment during IEMB operation might generate secondary pollution problems in the treated water and lead to the undesired growth of bacteria. Thus, for operation as a bioreactor, the membranes must assure a low permeability towards the employed carbon source, ethanol (selected as a non-charged electron donor) in this case. The TOC measurements performed during IEMB experiments showed that the migration of the carbon source and other metabolic by-products to the treated water was adequately avoided (i.e., TOC increase was not detected). To confirm this, additional tests on ethanol permeation were performed, and the results are shown in Figure 6.

From Figure 6, the diffusion coefficients of ethanol through the membranes are determined as 5·10^−8^ cm^2^ s^−1^ Amb-RE < 1·10^−7^ cm^2^ s^−1^ Pur-RE < 2·10^−7^ cm^2^ s^−1^ Lew-RE < 6·10^−7^ cm^2^ s^−1^ AMH-PES.

The advantageous lower diffusion coefficient of ethanol in Pur-RE, Amb-RE, and Lew-RE in respect to AMH-PES could be a consequence of the lower water content of the prepared membranes [6,43].

Overall, this work investigated for the first time the proof-of-concept validation of RO upcycled AEMs in DD and IEMB processes. Among the prepared membranes, Pur-RE shows the best performance in DD and IEMB operation as it combines the highest transmembrane flux of nitrate together with the best recovery yield in the receiver compartment (under DD operation), an adequately low co-ion leakage, and an undetectable transport of the carbon source and microbial metabolic by-products to the treated water compartment (under IEMB operation). Still, the lower transmembrane ion flux of Pur-RE in comparison with the reference commercial membrane could entail a limitation for its real application, and in this sense, future research should be dedicated to upgrading the performance of the membrane under DD and IEMB operating conditions. For that purpose, different strategies might be followed, either focused on improving membrane properties (e.g., by an activation treatment [5], using conductive nanomaterials as additives in the formulation [44,45], or increasing the selectivity towards the target counter ion [46]) or optimizing process parameters (e.g., increasing the relation between the membrane area per volume to be treated by using a plate and frame configuration [30], increasing the concentration of the stripping ion [47], or further avoiding co-ion leakage by choosing a divalent co-ion such as CaCl_2_ [8]).

### 3.3. 2D Fluorescence Spectroscopy

In this work, 2D fluorescence spectroscopy has been employed to gain a better understanding about the stability of the membranes against fouling and chemical cleaning. Such properties might be related to the lifetime of the membranes in a real application. In addition, understanding the fluorescence response of the membranes could help the implementation of an in situ monitorization of the bioreactors [35].

**Figure 7:**
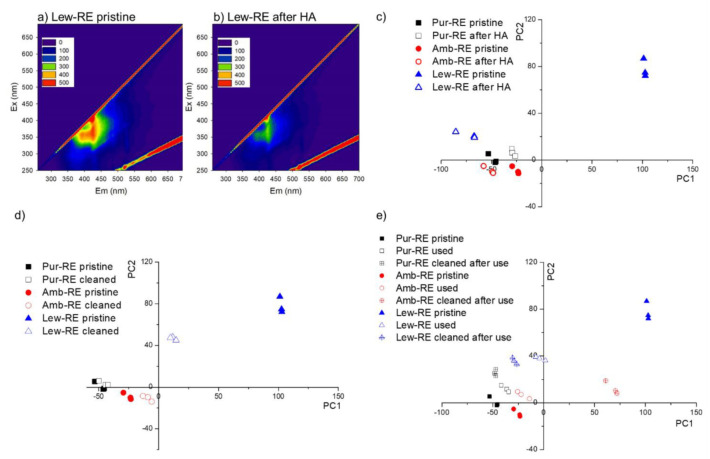
(**a**,**b**) Fluorescence spectra obtained from the coated (top) side of Lew-RE in pristine state (**a**) and after the accelerated fouling test (**b**); (**c**) scores for PC1 and PC2 obtained from the EEMs obtained from the coated (top) side of pristine membranes and membranes after the accelerated fouling test; (**d**) scores for PC1 and PC2 obtained from the EEMs obtained from the coated (top) side of pristine membranes and membranes after the chemical cleaning; (**e**) scores for PC1 and PC2 obtained from the EEMs of pristine membranes, used membranes (after the IEMB experiments), and cleaned membranes after their use in a first batch of experiments.

Due to the complexity of the EEMs obtained from the 2D fluorescence spectroscopic analysis, a Principal Components Analysis (PCA) was conducted to reduce the dimensionality of the data, while capturing the principal interrelations of the original data [37]. The PCA performed using all the data (i.e., Pur-RE, Amb-RE, Lew-RE, and AMH-PES) is displayed as Supplementary Material in Figure S3. Due to significant differences among the PCs of the reference (AMH-PES) and the prepared membranes, another PCA was performed including only the spectral data of the prepared membranes (i.e., Pur-RE, Amb-RE, and Lew-RE, excluding AMH-PES), aiming at a more precise analysis of the relation between the PCs of the prepared membranes. Results corresponding to the PCA analysis of the coated side of prepared membranes are displayed in Figure 7, while results corresponding to the PET side of the membranes are displayed as Supplementary Material in Figure S4.

Figure 7a,b shows the EEMs of Lew-RE membrane before and after the accelerated fouling test. It can be observed that the pristine Lew-RE membrane displays a high fluorescence intensity at the wavelength (λ) region λ_excitation_ = 320–420 nm/λ _emission_ = 350–450 nm (Figure 7a) while, after the accelerated fouling test, the excitation emission intensity is reduced (Figure 7b). This reduction in the emission intensity might be attributed to the deposition of humic acids on the membrane surface, partially absorbing the light emitted by the membrane and decreasing the emission intensity [32]. In respect to pristine membranes, it can be observed by the PCA that the PCs of the Lew-RE membrane differs from the PCs of Pur-RE and Amb-RE membranes, indicating differences between their EEMs. These results could be related to the different ion-exchange resins used in the preparation of the membranes, as they differ in the substituents at the ion-exchange sites (see Table 1), having different hydrophilic properties [6]. In contrast, the PET sides of the membranes were found to be rather similar to each other (Figure S4a, Supplementary Materials), as the ion-exchange resin is barely present at the PET side (confirmed by µ-Ramn analyses).

In respect to the development of a fouling layer, the PCA analysis (Figure 7c) shows a considerable distance between the PCs of the pristine and fouled Lew-RE membrane, indicating this membrane is the one that suffers higher alterations due to organic fouling.

Regarding the effect of the chemical cleaning on pristine membranes (Figure 7d), it can be observed that the PC scores of the pristine and cleaned Lew-RE membrane are the most distant from each other, indicating that Lew-RE membrane seems to be significantly affected by the employed chemical cleaning. In contrast, the Pur-RE membrane shows a negligible alteration of its fluorescence spectra.

It can be observed in Figure 7e that, on the one hand, the fluorescence response of the Lew-RE membrane becomes significantly altered after its use in the IEMB. In contrast, Pur-RE and Amb-RE membranes show a small alteration after their use. It should be noticed that in these experiments the biocompartment was faced to the PET surfaces of the membranes, and thus, the coated side should not be affected by biofouling. Therefore, the alteration shown by the Lew-RE membrane could be related to the adsorption of nitrate at the membrane surface, facilitated by the higher affinity of this membrane towards nitrate ions [6], as it was demonstrated before that nitrate can induce a quenching effect on the fluorescence spectra [48]. On the other hand, it can be observed in Figure 7e that the “pristine state” of the used membranes was not fully recovered after chemical cleaning. Indeed, the PCs are even more distant from the original (pristine state) after chemical cleaning. In the case of the Amb-RE membrane, the alteration induced by chemical cleaning on the used membrane seems to be more significant than in the case of the pristine membrane (results shown in Figure 7d). Again, the Pur-RE membrane is the one that suffers a less significant alteration under the tested conditions. In contrast, the PET side of Lew-RE membrane shows the lowest alteration under these conditions (information detailed in Figure S4c, Supplementary Materials).

In conclusion, it can be stated that the variations in the excitation emission spectra are well captured by the first two PCs in the performed PCA analysis. Concerning the resistance to alterations caused by organic fouling and the exposure to acid and alkali solutions, the Lew-RE membrane shows to be the least resistant, while the Pur-RE membrane appears to be the most stable one, under the tested conditions.

As a complementary study, an Attenuated total reflectance–Fourier transform infrared spectroscopy (ATR-FTIR) analysis of the membranes in pristine state and after the accelerated fouling test and the chemical cleaning of pristine membranes was conducted (see Section S4 of the Supplementary Materials). Interestingly, differences in the functional groups of the membranes (after the accelerated fouling test and the chemical cleaning) were not detected, indicating that the mentioned conditions were not harsh enough to induce chemical alterations perceived by this technique. Accordingly, it is worthy to remark that, through the fluorescence response studies performed, it was possible to notice differences in the chemical composition of the membranes’ surfaces, after the accelerated fouling test and the chemical cleaning, indicating therefore a high sensitivity of 2D fluorescence spectroscopy to detect subtle alterations at the membrane surfaces.

### 3.4. Preliminary Membrane-Associated Costs and Environmental Implications

The aim of this section is to provide a preliminary estimation, for the first time, of the approximated material cost of Pur-RE membrane. In addition, the economic affordability of Pur-RE membrane and reference commercial IEM (AMH-PES) were compared, taking into consideration the differences in ion transport performances between the membranes. In light of the preliminary nature of this proof-of-concept study, CAPEX and OPEX considerations in relation to the industrial application of DD and IEMB systems are out of the scope of this work. The cost of recycling an end-of-life brackish water RO membrane into UF-like properties, including capital expenditure (CAPEX) and the operational expenditure costs (OPEX), has been estimated in a previous study [49], as EUR 31.7 m^−2^ per module. Considering an active membrane area of 37 m^2^ per module, the cost of the recycled membrane support could approach EUR 0.9 m^−2^. In addition, the cost of the coating layer, regarding the current price in market of the employed reagents (displayed in Table S2 as Supplementary Material) and excluding CAPEX and OPEX considerations, could approach approximately EUR 2.4 m^−2^. Under these considerations, the costs of the materials required to prepare the Pur-RE membrane could approach EUR 3.3 m^−2^. It is worth noting that this preliminary estimation does not consider the costs associated with module opening and repacking, which should be evaluated in future studies. For comparison, the selling price of AMH-PES Ralex ^®^ membrane is between EUR 70 and 100 m^−2^ [50]. Since transmembrane NO_3_^−^ flux through Pur-RE membrane was lower than in the case of the reference commercial membrane, for the sake of a roughly preliminary comparison, Table 2 shows the equivalent surface of Pur-RE membrane to reach the same removal rate of AMH-PES membrane. Two different scenarios have been considered, the first one calculating the equivalent membrane surface of Pur-RE considering the transmembrane ion flux to reach nitrate concentration below the maximum allowed (50 ppm), and a second one considering the transmembrane ion flux to reach a level below the recommended nitrate concentration (25 ppm). The transmembrane fluxes were calculated based on the data reported in Section 3.2.1.

Certainly, a deeper economic assessment should be conducted (e.g., the cost of module opening and repacking should be evaluated, and CAPEX and OPEX should be considered), as well as an improvement in the production process (e.g., reducing the costs associated to the coating process, while improving the ion transport performance of the resulting membranes), but even in that case, it seems evident a significant economic benefit if this concept is brought to market. In addition, the European green policies for a sustainable transition (i.e., the European Green Deal [51] and the Circular Economy Action Plan [52]) advocate giving rise to taxation on landfill disposal, encouraging the extension of the service time of goods and products and making recycling alternatives more attractive from an economic point of view.

From an environmental point of view, the recycling membrane concept presented in this study is an alternative to conventional ion-exchange membrane production. In this sense, in a previous study [49], savings of more than 175 kg CO_2_ equivalent and 0.28 m^3^ of freshwater (among other environmental indicators) were estimated trough the Life Cycle Assessment (LCA) of the recycling process of one end-of-life RO module into UF-like properties. Therefore, even if CO_2_ equivalent emission, related to the coating process, is not yet accounted for, it is possible to envisage a positive environmental impact of the proposed recycling concept.

Overall, membrane recycling is a promising alternative to waste disposal by RO industry and it is a commitment to circular economy in membrane technology and the water sector. Furthermore, it has been demonstrated that passive mass transport processes such as DD and IEMB could be a promising technological niche for the application of such AEMs.

## 4. Conclusions

In the present work, the feasibility of using AEMs prepared from upcycled end-of-life RO membranes for the removal of nitrate from water using DD and IEMB systems is demonstrated. The main findings of the study are summarized as follows:

1. µ Raman spectroscopy confirmed the successful deposition of the ion-exchange resin in the coated (top) surface of the membrane. However, the analysis revealed an asymmetric distribution of the ion-exchange sites in the membrane cross-section, which might contribute to the relatively low ion-exchange capacity and water content and the relatively high electrical resistance of the membranes and, therefore, have a great influence on transport properties of the membrane.
2. In respect to nitrate removal under DD and IEMB operation, the membrane incorporating Purolite^®^ A600/9149 ion-exchange resin (Pur-RE membrane) achieved, among the prepared membranes, the best removal yields (57% and 56% of nitrate removal within 24 h under DD and IEMB operation, respectively), probably due to its higher ion-exchange capacity and lower electrical resistance, in comparison with the Amb-RE and the Lew-RE membranes. Furthermore, nitrate was biologically eliminated in the IEMB, favouring the reuse of the receiver solution and avoiding the discharge of the pollutant into the environment.
3. 2D fluorescence spectroscopy can effectively detect alterations in the excitation emission spectra of the membranes, caused by fouling and/or chemical cleaning. In this respect, the Pur-RE membrane was found to be the most stable membrane under the tested conditions.
4. The relative low cost of the employed materials in the formulation of Pur-Re membrane anticipated an economic benefit of the presented membrane recycling concept.

Overall, this work shows the potential applicability of RO upcycled membranes as AEMs in electrochemical potential driven processes (DD and IEMB), for an efficient removal of nitrate from water at a minimum energy requirement. The developed membranes could be obtained at a lower cost than commercial ones and therefore facilitate the practical implementation of these passive transport processes. Lastly, membrane recycling represents a commitment to the transition to a circular economy.

## Figures and Tables

**Table 1:** Main characteristics of the membranes under study. Thickness, water content, Ion-Exchange Capacity (IEC), electrical resistance, and permselectivity of all the membranes were experimentally measured under the same conditions and previously reported (data from [6]). AMH-PES membrane composition is detailed by the provider (data from [29]).

Anion-Exchange Membrane	AMH-PES	Pur-RE	Amb-RE	Lew-RE
Mechanical support	Polyester (PET)	End-of-life RO	End-of-life RO	End-of-life RO
Polymer binder	Polyethylene (PE)	PVC	PVC	PVC
Ion-exchange resin	Unspecified	Purolite^®^ A600/9149	Amberlite^®^ IRA-402	Lewatit^®^ Sybron Ionac^®^ SR-7
Ion-exchange group	R—(CH_3_)_3_N^+^	R—(CH_3_)_3_N^+^	R—(CH_3_)_3_N^+^	R—(C_3_H_7_)_3_N^+^
Membrane thickness (µm)	645 ± 5	190 ± 4	184 ± 7	182 ± 7
Water content (%) ^a^	50 ± 0	23 ± 4	21 ± 4	19 ± 1
IEC (mmol g^−1^) ^b^	2.19 ± 0.09	0.75 ± 0.14	0.62 ± 0.04	0.39 ± 0.01
Permselectivity (%) ^c^	81 ± 1	65 ± 9	83 ± 7	66 ± 4
Electrical resistance (Ω cm^2^) ^d^	19 ± 3	56 ± 7	129 ± 1	120 ± 11

^a^ Gravimetric method. ^b^ Immersed in KNO_3_ 1 M for 24 h under stirring (100 rpm); immersed in NaCl 0.5 M for 24 h under stirring (100 rpm); UV-VIS spectrophotometric determination of the nitrate released in NaCl solution. ^c^ Measured in 0.1 and 0.5 M NaCl solutions. ^d^ Measured in 0.5 M NaCl solution.

**Table 2:** Approximated cost of Pur-RE considering the differences between the target ion transmembrane flux $\left(J_{NO_{3}^{-}}\right)$ in respect to the reference membrane (AMH-PES). $J_{NO_{3}^{-}\;\left(C_{0}-C_{50\;}\right)}$, nitrate transmembrane flux from the initial conditions to reach a concentration below the maximum allowed level (50 mg L^−1^), $J_{NO_{3}^{-}\;\left(C_{0}-C_{25}\right)}$, nitrate transmembrane flux from the initial conditions to reach a concentration below the maximum recommended level (25 mg L^−1^).

$\mathrm{Transmembrane}\;{{\mathrm{NO}}_{3}}^{-}\;\mathrm{Flux}\;\left(J_{NO_{3}^{-}}\right)$	Pur-RE	AMH-PES	Pur-RE Membrane Surface (m^2^) Equivalent to Performance of 1 m^2^ AMH-PES *	Pur-RE Membrane Cost (EUR) Equivalent to Performance of 1 m^2^ AMH-PES **
$J_{NO_{3}^{-}\;\left(C_{0}-C_{50\;}\right)}$ (g m^−2^ h^−1^) in DD	0.82	6.34	7.73	25.2
$J_{NO_{3}^{-}\;\left(C_{0}-C_{25\;}\right)}$ (g m^−2^ h^−1^) in DD	0.18	2.70	14.81	48.3
$J_{NO_{3}^{-}\;\left(C_{0}-C_{50\;}\right)}$ (g m^−2^ h^−1^) in the IEMB	0.81	6.00	7.39	24.1
$J_{NO_{3}^{-}\;\left(C_{0}-C_{25\;}\right)}$ (g m^−2^ h^−1^) in the IEMB	0.19	2.61	13.50	44.0

* Considering the data in Section 3.2.1. ** Considering the estimated material cost for Pur-RE as 3.3 EUR m^−2^.

## Supplementary Materials

The following supporting information can be downloaded at https://www.mdpi.com/article/10.3390/membranes12020101/s1. Table S1: Main characteristic Raman bands (cm^−1^), between 200–2000 cm^−1^, for Polyvinyl chloride (PVC), the ion exchange resin (Purolite^®^ A600/9149), Polysulfone (PSf), and Polyester (PET), identified in the membrane Pur-RE. Figure S1: µ-Raman spectra between 200–2000 cm^−^^1^ of polyvinyl chloride (PVC), the ion exchange resin (Purolite^®^ A600/9149), polysulfone (PSF), and polyester (PET), identified in the membrane Pur-RE. Figure S2: Time course concentration of nitrite ([NO_2_^−^]) in IEMB experiments (a) feed compartment and (b) receiver compartment. Membranes: Pur-RE, Amb-RE, Lew-RE, and AMH-PES. Figure S3. Scores for PC1 and PC2 obtained from the PCA of all EEMs of the tested membranes. Side a and side b in AMH-PES refers to the membrane surfaces in contact with the feed and the receiver compartments, respectively. Figure S4: Scores for PC1 and PC2 obtained from the EEMs of the PET surface in pristine membranes and (a) membranes after the accelerated fouling test, (b) membranes after the chemical cleaning, and (c) membranes after their use in the bioreactor (i.e., after the IEMB experiments) and posterior cleaning. Figure S5: ATR-FTIR spectra of studied membranes in pristine state, after the accelerated fouling test, and after the chemical cleaning treatment cleaning, (a) Pur-RE, (b) Amb-RE, (c) Lew-RE, (d) AMH-PES. Table S2: Calculation of the price of consumable materials for the preparation of 1 m^2^ of AEM using the methodology described in Section 2.2. of the manuscript. References [4,23,38,53,54,55,56] are cited in the Supplementary Materials.
