# Supplementary material for: Nitrate Removal by Donnan Dialysis and Anion-Exchange Membrane Bioreactor Using Upcycled End-of-Life Reverse Osmosis Membranes
Source: Membranes (Basel). 2022 Jan 18;12(2):101. doi: 10.3390/membranes12020101 (PMC8878892; doi:10.3390/membranes12020101)
Supplement: Supplementary file 1 [file membranes-12-00101-s001.zip › membranes-1535949-supplementary.pdf]

Supplementary Material

for

Nitrate Removal by Donnan Dialysis and Anion-Exchange Membrane Bioreactor Using Upcycled End-of-life Reverse Osmosis Membranes

Amaia Lejarazu-Larrañaga [1, 2, *], Juan M. Ortiz [1], Serena Molina [1, *], Sylwin Pawlowski [3], Claudia F. Galinha[3], Vanessa Otero [4, 5], Eloy García-Calvo [1, 2], Svetlozar Velizarov [3], João G. Crespo [3].

[1] IMDEA Water Institute, Avenida Punto Com, 2, 28805. Alcalá de Henares, Madrid, Spain

[2] Chemical Engineering Department, University of Alcalá, Ctra. Madrid-Barcelona Km 33.600, 28871. Alcalá de Henares, Madrid, Spain

[3] LAQV-REQUIMTE, Department of Chemistry, NOVA School of Science and Technology, FCT NOVA, Universidade NOVA de Lisboa, 2829-516 Caparica, Portugal

[4] LAQV-REQUIMTE, Department of Conservation and Restoration, NOVA School of Science and Technology, FCT NOVA, Universidade NOVA de Lisboa, 2829-516 Caparica, Portugal

[5] VICARTE, Department of Conservation and Restoration, NOVA School of Science and Technology, FCT NOVA, Universidade NOVA de Lisboa, 2829-516 Caparica, Portugal

Correspondence to:

amaia.ortiz@imdea.org

serena.molina@imdea.org

S1. Confocal micro-Raman spectroscopy

Table S1 shows the main Raman bands identifying the polymers in the membrane.

Table S1. Main characteristic Raman bands (cm^{-1}), between 200-2000 cm-1, for Polyvinyl chloride (PVC), the ion exchange resin (Purolite® A600/9149), Polysulfone (PSf) and Polyester (PET), identified in the membrane Pur-RE [3].

PVC	Ion exchange resin	PSf	PET	Vibrational assignments
638				ν(C-Cl)
695				ν(C-Cl)
	720			Ring δ(CH)
	764	792		Ring breathing
			858	Ring δ(CH)
		1109		ν(SO_2)
		1150		ν(C-O-C)
	1190			Ring ν(CH)
	1221			Ring ν(CH)
			1288	ν(C(=O)-O)
1428				δ(CH_2)
		1589		Ring ν(CC)
	1612	1609	1616	Ring ν(CC)
			1727	ν(C=O)

Fig. S1 shows the μ-Raman spectra identifying different polymers present in the Pur-RE membrane.

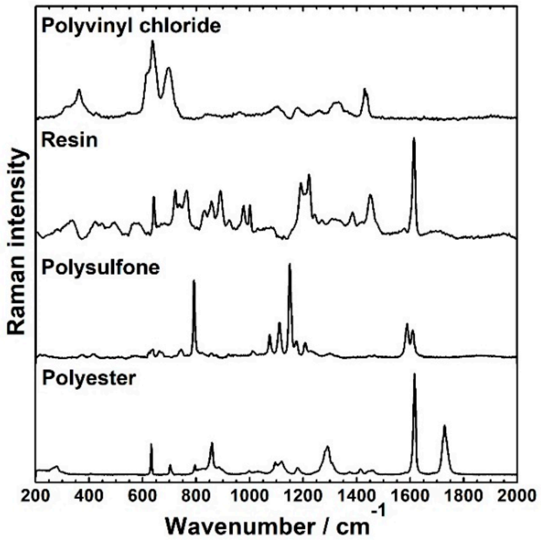

Figure S1. μ-Raman spectra between 200-2000 $cm^{-1}$of polyvinyl chloride (PVC), the ion exchange resin (Purolite® A600/9149), polysulfone (PSF) and polyester (PET), identified in the membrane Pur-RE.

S2. Ion-exchange membrane bioreactor (IEMB) experiments

Fig. S2 shows the measured concentration of nitrite (NO_2^-) in the feed and in the receiver compartments during IEMB experiments.

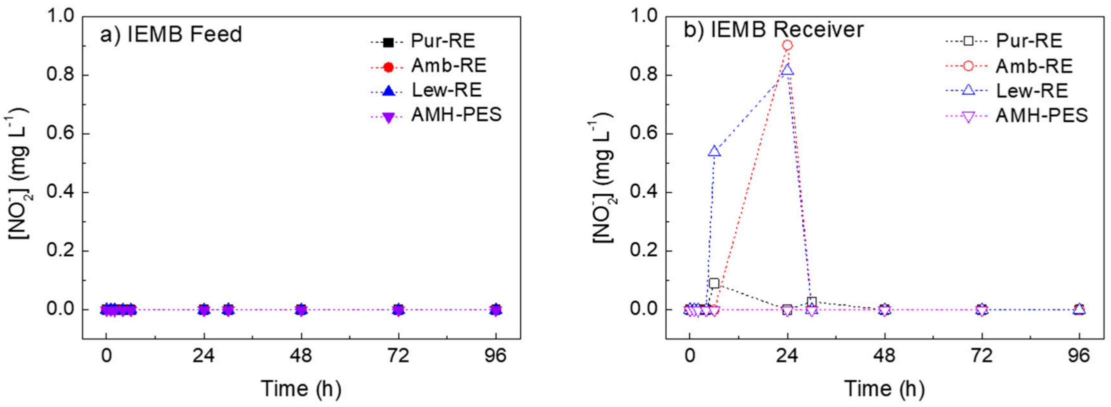

Figure S2. Time course concentration of nitrite ($[NO_2^-]$) in IEMB experiments a) feed compartment, and b) receiver compartment. Membranes: Pur-RE, Amb-RE, Lew-RE, AMH-PES.

It can be observed that nitrite was not detected at any time in the feed compartment. In the receiver compartment, appearance of the intermediate nitrite trace accumulation was detected (at concentrations below 1 mg L^{-1}), which was completely eliminated as the experiment progressed.

S3. 2D fluorescence spectroscopy.

PCA including Pur-RE, Amb-RE and Lew-RE and AMH-PES

Fig. S3 shows the correlation between the first two Principal Components (PCs) obtained from the PCA analysis performed including all the tested membranes (Pur-RE, Amb-RE, Lew-RE and AMH-PES).

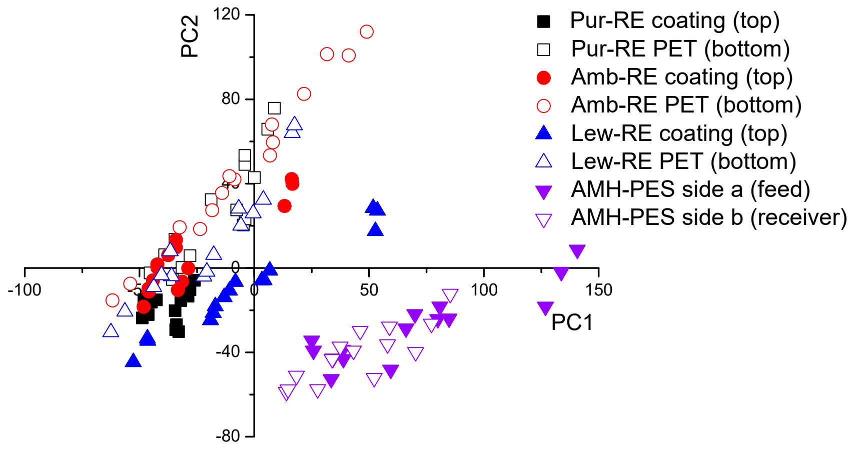

Figure S3. Scores for PC1 and PC2 obtained from the PCA of all EEMs of the tested membranes. Side a and side b in AMH-PES refers to the membrane surfaces in contact with the feed and the receiver compartments, respectively.

With these PCs, two different data clusters can be well differenced, one corresponding to the prepared membranes (Pur-RE, Amb-RE and Lew-RE), and another one corresponding to the commercial membrane (AMH-PES). Even if those clusters are well divided by the PCs, they are

not completely unrelated as the data in both clusters follows a similar direction [4]. Interestingly, differences between the coating layer and the PET bottom surface of the prepared membranes can be distinguished, as a reflect of the asymmetric membrane structure, confirmed by confocal μ-Raman spectroscopy.

Differences between Pur-RE, Amb-RE and Lew-RE membranes at the PET surface.

Figure S4 shows the correlation between the first two first PCs of the EEMs obtained at PET surface of the prepared membranes in pristine state, after the fouling test, after the chemical cleaning treatment and after their use in the bioreactor and a posterior cleaning.

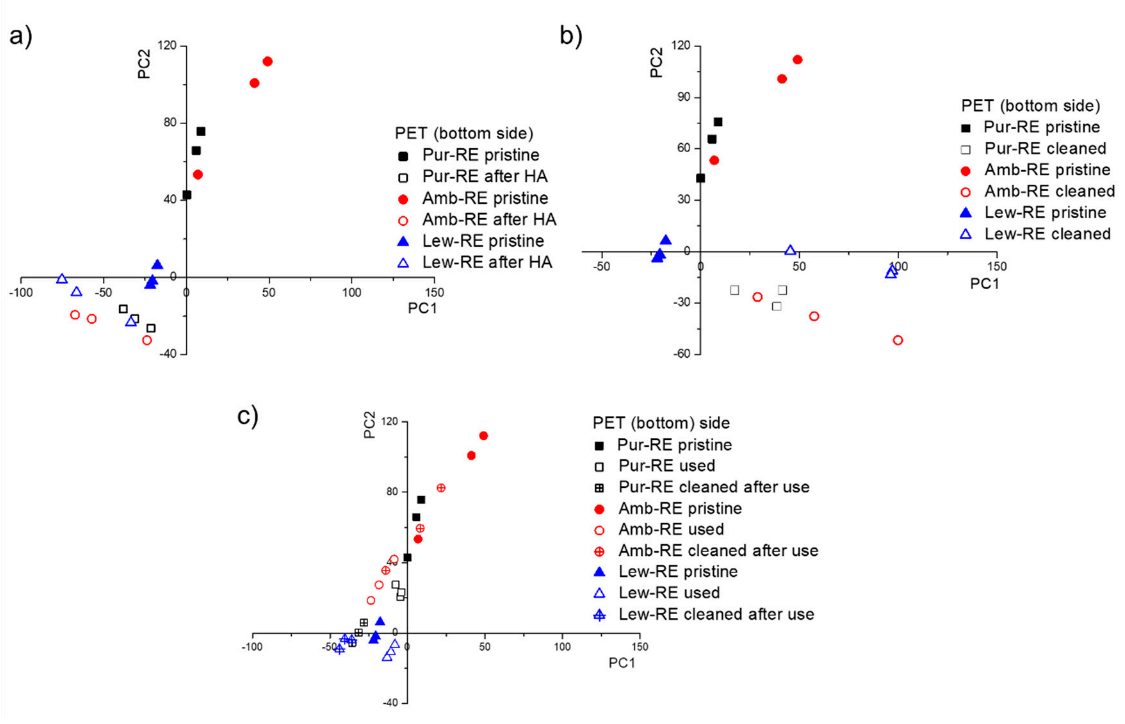

Figure S4. Scores for PC1 and PC2 obtained from the EEMs of the PET surface in pristine membranes, and a) membranes after the accelerated fouling test, b) membranes after the chemical cleaning and c) membranes. After their use in the bioreactor (i.e., after the IEMB experiments) and posterior cleaning.

As expected, PCA analyses found similarities between the PET (bottom) surfaces in pristine membranes (i.e., PC scores aligned in the same direction), due to the fact that the ion exchange resin (different in each membrane) is not present at this side of the membrane (confirmed μ-Raman spectroscopy).

Regarding the alterations of the fluorescence spectra caused by the fouling treatment (Fig S4a), it can be stated that HA fouling affects in a greater extent the PET surface of the membranes in comparison with the coating layer, which could be attributed to the lower hydrophilicity of the PET fabric [5].

In addition, in Fig S4b it can be observed that the cleaning treatment affected in a similar way to all the tested membranes, causing significant alterations on the EEMs of the PET surfaces, in respect to the pristine state.

Lastly, in Fig. S4c, it can be observed that, as expected, the use of the membranes in the bioreactor affected to the fluorescence response of the PET (bottom) surfaces of the membranes (in contact with the biocompartment during the experiments). In contrast to the results at the coated (top) surface of the membrane, the PET (bottom) surface of Lew-RE membrane shows the lowest alteration of the fluorescence response. In this case, the hydrophobic properties of Lewatit ® Sybron Ionac ® SR-7 ion-exchange resin could lead to the penetration of HA-like compounds into the PET side of the membranes and their transport to the coated (top) side. Causing a greater alteration at the coated (top) side than at the PET (bottom) side of the membrane.

S4. Attenuated total reflectance–Fourier transform infrared spectroscopy (ATR-FTIR)

Fourier transform infrared (FTIR) measurements were carried out using a spectrometer (Perkin-Elmer, Waltham, MA, USA) coupled with an attenuated total reflection (ATR) device. Sixteen scans were averaged from 4000 to 650 cm^{-1} and with a resolution of 4 cm^{-1}. The effect of the accelerated fouling test and the cleaning treatment on the functional groups of pristine membranes was studied. For that purpose, Pur-RE, Amb-RE, Lew-RE and AMH-PES membranes in pristine state and after the mentioned treatments were analysed. The membranes were dried in an oven at 50ºC prior to the analysis.

Fig. S5 shows the ATR-FTIR spectra of the analysed membranes in pristine state, after the accelerated fouling test, and after chemical cleaning of pristine membranes.

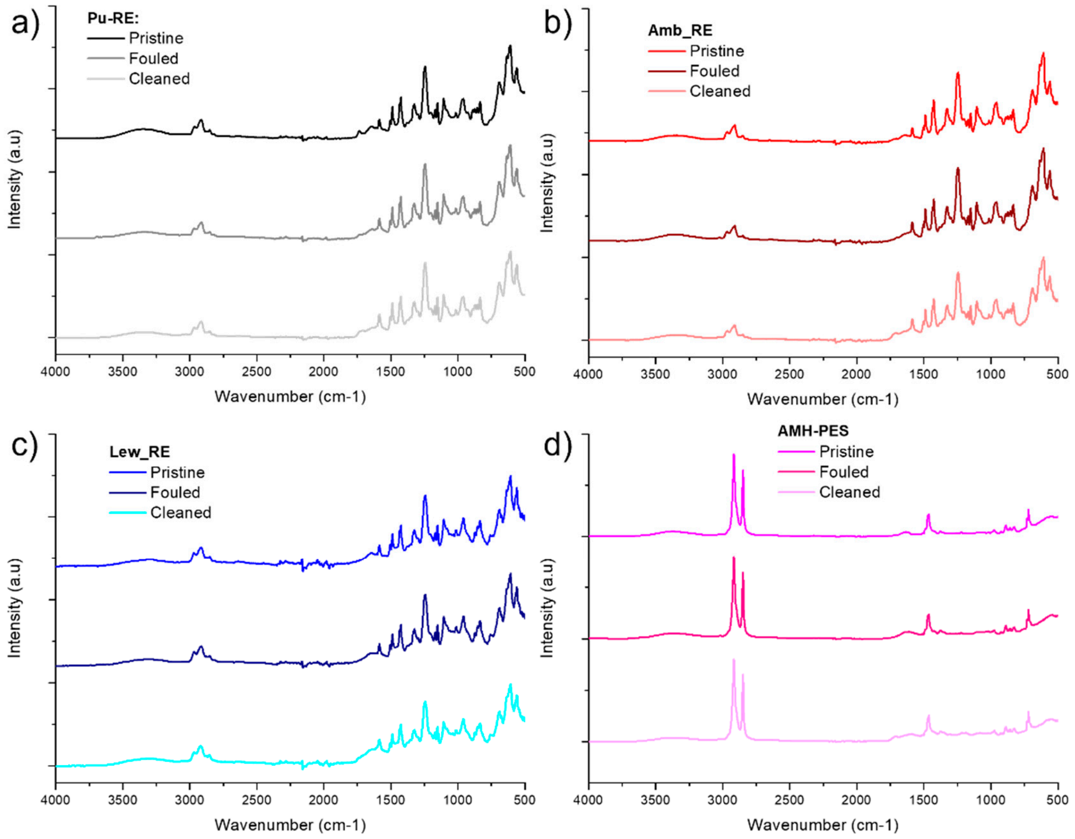

Figure S5. ATR-FTIR spectra of studied membranes in pristine state, after the accelerated fouling test and after the chemical cleaning treatment cleaning, a) Pur-RE, b) Amb-RE, c) Lew-RE, d) AMH-PES.

It can be observed in Fig. S5 (a to d) that the ATR-FTIR spectra of the membranes after the accelerated fouling test and the chemical cleaning remain unchanged in respect to the pristine state. These results indicate that these treatments did not induce significant chemical alterations in the functional groups of the membranes. In contrast, the fluorescence responses of the membranes were significantly altered, as it was stated by 2D fluorescence spectroscopy analysis (sections 3.3 and S3).

S5. Estimation of the material cost of the coating layer

Table S2. Calculation of the price of consumable reagents used in the coating layer for the preparation of 1 m^2 AEM, according to the methodology described in section 2.2. of the manuscript.

Consumable materials		€ per unit	Amount per m^{-2} AEM	Price per m^2 AEM (€ m^{-2})	Reference
Organic solvent	Tetrahydrofuran (THF)	5.36 – 5.43 € L^{-1} [a]	0.40 L	2.16	[6]
Polymer binder	Polyvinylchloride (PVC) powder	0.70 – 0.72 € kg^{-1} [b]	0.0198 kg	0.01	[7]
Anion Exchange Resin	Purolite ® A600/9149	6.74 € kg^{-1} [c]	0.0361 kg [d]	0.24	Purolite®®

[a] 36500-3700 yuan per metric ton (data referred to 2021), considering a density of 0.889 kg m^{-3} (at 25ºC).
[b] 835-850 U.S. dollar per metric ton (data referred to 2020-2022).
[c] 168.75 € per 25 kg (data referred to 2021).
[d] Considering a weight loss of 45% of the product during drying stage before membrane preparation.

References

1. Lejarazu-Larrañaga, A.; Molina, S.; Ortiz, J.M.; Navarro, R.; García-Calvo, E. Circular economy in membrane technology: Using end-of-life reverse osmosis modules for preparation of recycled anion exchange membranes and validation in electrodialysis. *J. Memb. Sci.* **2020**, *593*, 117423, doi:10.1016/j.memsci.2019.117423.
2. Rodríguez-Sáez, L.; Landaburu-Aguirre, J.; Molina, S.; García-Payo, M.C.; García-Calvo, E. Study of surface modification of recycled ultrafiltration membranes using statistical design of experiments. *Surfaces and Interfaces* **2021**, *23*, doi:10.1016/j.surfin.2021.100978.
3. Hummel, D.O. *Atlas of Plastics Additives. Analysis by Spectrometric Methods*; Springer-Verlag: Berlin, 2002;
4. Galinha, C.F.; Carvalho, G.; Portugal, C.A.M.; Guglielmi, G.; Oliveira, R.; Crespo, J.G.; Reis, M.A.M. Real-time monitoring of membrane bioreactors with 2D-fluorescence data and statistically based models. *Water Sci. Technol.* **2011**, *63*, 1381–1388, doi:10.2166/wst.2011.195.
5. Zaman, M.; Liu, H.; Xiao, H.; Chibante, F.; Ni, Y. Hydrophilic modification of polyester fabric by applying nanocrystalline cellulose containing surface finish. *Carbohydr. Polym.* **2013**, *91*, 560–567, doi:10.1016/j.carbpol.2012.08.070.
6. Tetrahydrofuran Market Price & Analysis Available online: https://www.echemi.com/productsInformation/pd20150901266-tetrahydrofuran.html (accessed on Sep 7, 2021).
7. Statista Price of polyvinyl chloride worldwide from 2017 to 2020 with estimated figures for 2021 to 2022 Available online: https://www.statista.com/statistics/1171131/price-polyvinyl-chloride-forecast-globally/#statisticContainer (accessed on Sep 6, 2021).
